# Mechanistic Insights of Plant Growth Promoting Bacteria Mediated Drought and Salt Stress Tolerance in Plants for Sustainable Agriculture

**DOI:** 10.3390/ijms23073741

**Published:** 2022-03-29

**Authors:** Anmol Gupta, Richa Mishra, Smita Rai, Ambreen Bano, Neelam Pathak, Masayuki Fujita, Manoj Kumar, Mirza Hasanuzzaman

**Affiliations:** 1 IIRC-3, Plant–Microbe Interaction and Molecular Immunology Laboratory, Department of Biosciences, Faculty of Science, Integral University, Lucknow 226026, Uttar Pradesh, India; anmolgupta632@gmail.com (A.G.); shiblyrai@gmail.com (S.R.); ambreenbano2408@gmail.com (A.B.); 2 Department of Biochemistry, Dr. Rammanohar Lohia Avadh University, Ayodhya 224123, Uttar Pradesh, India; richa7087@gmail.com (R.M.); pathak.neelam007@gmail.com (N.P.); 3 Laboratory of Plant Stress Responses, Faculty of Agriculture, Kagawa University, Kagawa 761-0795, Japan; 4 Institute of Plant Sciences, Agricultural Research Organization, Volcani Center, Rishon LeZion 7505101, Israel; 5 Department of Agronomy, Faculty of Agriculture, Sher-e-Bangla Agricultural University, Dhaka 1207, Bangladesh

**Keywords:** antioxidant defense, biostimulants, osmotic stress, plant–microbe interaction, reactive oxygen species, water deficit

## Abstract

Climate change has devastating effects on plant growth and yield. During ontogenesis, plants are subjected to a variety of abiotic stresses, including drought and salinity, affecting the crop loss (20–50%) and making them vulnerable in terms of survival. These stresses lead to the excessive production of reactive oxygen species (ROS) that damage nucleic acid, proteins, and lipids. Plant growth-promoting bacteria (PGPB) have remarkable capabilities in combating drought and salinity stress and improving plant growth, which enhances the crop productivity and contributes to food security. PGPB inoculation under abiotic stresses promotes plant growth through several modes of actions, such as the production of phytohormones, 1-aminocyclopropane-1-carboxylic acid deaminase, exopolysaccharide, siderophore, hydrogen cyanide, extracellular polymeric substances, volatile organic compounds, modulate antioxidants defense machinery, and abscisic acid, thereby preventing oxidative stress. These bacteria also provide osmotic balance; maintain ion homeostasis; and induce drought and salt-responsive genes, metabolic reprogramming, provide transcriptional changes in ion transporter genes, etc. Therefore, in this review, we summarize the effects of PGPB on drought and salinity stress to mitigate its detrimental effects. Furthermore, we also discuss the mechanistic insights of PGPB towards drought and salinity stress tolerance for sustainable agriculture.

## 1. Introduction

Plants have been increasingly vulnerable to environmental stresses due to climate change during ontogenesis [1], and the rising global mean temperature poses a serious threat to agriculture and socioeconomic development [2]. Climate change globally causes a variety of environmental stresses, which are damaging to agricultural crop production. Environment perturbations that cause metabolic disruption, development, and yield disruption are considered a stress situation during their ontogenesis and cause stress reactions. Stress situations in biological systems are characterized by significant perturbations to the metabolism, development, and yield during their ontogenesis. The natural environment is filled with biotic and abiotic stresses that negatively impact plant growth and productivity [3,4,5]. Among abiotic stresses, salinity and drought account for 50% of productivity losses [6]. The Food and Agricultural Organization (FAO) reported that the annual agricultural productivity losses range between 20 and 40%, totaling US $31 million due to salt stress [7]. At the global level, abiotic stresses such as drought and salinity adversely affect crop output, even though they manifest differently depending on the region [8]. As abiotic stresses are interrelated and often occur together in a natural environment, they have especially severe effects on plants, impacting their cellular, metabolic, and physiological activities and reducing crop yields [1,9]. Abiotic stress may affect the Calvin cycle, photosystems (PS) or photosynthetic enzymatic activity, stomatal function, and even alter electron transport chain (ETC) reactions [10].

The growth, development, and fertility of plants are rigorously influenced by drought stress. During salt and drought conditions, plants respond similarly in physiological and molecular ways, including osmotic imbalance, cell dehydration, and the production of ROS [11,12]. Drought causes water loss and reduces the water potential, which, in turn, reduces cell turgor [13]. Abscisic acid (ABA)-mediated stomata closure is particularly the fastest process induced by drought [14]. Extended drought stress leads to osmotic regulation [15,16], metabolic reprogramming [17], which leads to an accumulation of primary and secondary metabolites [18,19], a decreased root–shoot ratio [20], activation of the antioxidant system [8,21], and cell wall modifications [22,23]. The modifications of these traits can be measured and used to gauge the rigorousness of drought. The soil is classified as saline when its electrical conductivity (EC) is greater than four dS m^−1^ (approximately 40-mM NaCl) and has an exchangeable sodium content of 15% [24,25]. Soil salinity negatively influences agricultural productivity and soil fertility as well [9]. Additionally, drought stress and salinity affect the photosynthesis, plant transpiration, and functioning of roots [26]. Moreover, because of diverse factors, such as poor irrigation practices, climate alteration, and additional natural processes, croplands are degraded by drought and salinity by about 10% on an annual basis [9,14,20]. Drought stress induces stomatal closure and loses membrane integrity, while salinity stress can cause sodium (Na^+^) and chloride (Cl^−^) ion accumulation, thereby reducing plant growth and crop yields [11,12].

Around 1 to 2% of the oxygen (O_2_) utilized by plants is changed into reactive oxygen species (ROS), particularly singlet oxygen (^1^O_2_), hydroxyl radical (OH^•^), superoxide radical (O_2_^•−^), hydrogen peroxide (H_2_O_2_), alkoxyl radicals (RO^•^), peroxy radical (ROO^•^), and reactive nitrogen species (RNS), as a byproduct of aerobic metabolism in numerous cell organelles, like mitochondria, chloroplasts, nucleolus, and apoplasts [10,15]. Nevertheless, peroxisomes are also recognized to be powerful ROS generators, because photochemical reactions and ETC contribute the most of ROS production [27,28,29,30]. In plant cells, ROS tend to remain at a low level due to the antioxidant systems that regulate them. The rate of ROS production increases exponentially under conditions of drought and salinity stress, exceeding the capacity of antioxidant scavengers and causing an oxidative burst that interrupts the cellular redox homeostasis, affects biomolecules, and modulates the cellular mechanism, resulting in a negative balance between the production and scavenging of these reactive species [31,32,33]. Conversely, controlled ROS production contributes to redox signaling, plant–microbe interactions [28,29], plant growth, and its development under abiotic stress [27,34,35,36]. Occasionally, ROS can damage molecular and cellular components (like nucleic acids, including DNA and RNA and proteins) by oxidizing biomolecules (such as carbohydrates, proteins, lipids, enzymes, and DNA), which can lead to severe plant death [27]. Despite this, the exact mechanisms of ROS-mediated stress alleviation remain unclear. To avoid damage and cell death, the elevated production of ROS and RNS should be suppressed by antioxidant machinery.

Plants employ enzymatic and nonenzymatic antioxidant systems to cope with the damage caused by ROS production. The physiology and metabolism of plants may change either reversibly or irreversibly as a result of abiotic stresses [37]. Several enzymatic antioxidant systems, including catalase (CAT), superoxide dismutase (SOD), peroxidase (POX), ascorbate peroxidase (APX), lipid peroxidase (LPX), glutathione peroxidase (GPX), glutathione reductase (GR), etc., and nonenzymatic antioxidants like vitamins, tocopherols, stilbenes, phenols, ascorbate, glutathione, flavonoids, and carotenoids quench the excess ROS, thereby protecting cells from oxidative stress [20,22,38,39,40,41,42,43]. In fact, all plants possess these mechanisms, which can be referred to as “innate tolerance”. Aside from these forms of responses, certain plants, in comparison to others, have evolved the capacity to thrive under stressful situations. This response is commonly known as the “memory of stress” in plants and can be viewed as an “acquired tolerance”. In recent decades, flavonoids have been extensively debated as potent antioxidants both in plants and animals [40,44,45,46,47,48]. Despite the fact that flavonoids have the ability to mitigate the negative consequences associated with the enormous formation of ROS, authoritative criticism has been raised in a number of circumstances. ROS intermediates affect the PS and induce programmed cell death (PCD) [49] and are accountable for osmotic stress [50,51,52]. Plants have the ability to adapt salt and drought stress in different ways [13,53,54,55,56,57]. In addition, salinity and drought concentrations can decrease the efflux of macro and micronutrients (such as P, N, Mg, Fe, Cu, and Zn), which reduces their solubility and competition with Na^+^ and Cl^−^ [58,59]. Plant development is thus disturbed in various ways, such as germination, vegetative growth, and reproduction [41,60]. Due to this rising issue, it is appropriate to search for potential methodologies for increasing crop yields under drought and salinity. In contrast, several research studies highlighted the differences between salinity and drought, where both stresses can affect physiology and metabolism differently while, in many pathways, they act similar [61]. Numerous beneficial plant growth-promoting bacteria (PGPB) have been discovered by various researchers that have a positive impact on promoting plant growth by various mechanisms such as phytohormones production, ESP production, regulating the nutrient exchange and internal ionic content, and facilitating the biosynthesis of osmoprotectant compounds (e.g., total soluble sugar (TSS), proline, betaine, or trehalose, etc.) that reduce osmotic stress [18,40,41,62,63,64]. Despite the studies conducted to date on these mechanisms, there are still additional useful impacts of soil microbiota that have not been identified, underscoring the need for further research to optimize PGPB use in agricultural systems. Furthermore, it is imperative to remember that each of these mechanisms is interconnected, but even the same microorganism can have a diverse effect on the same plant. Thus, PGPB might be a useful strategy for boosting plant development and production in drought and salinity-stressed environments. The current review examines the existing literature about the impact of drought and salinity stresses on plant fitness, along with exploring antioxidant-based defense mechanisms that regulate ROS accumulation and reduce oxidative stress. Further, the molecular mechanisms of ROS generation in plants and cost-effective and eco-sustainable PGPB approaches in ameliorating abiotic stress have also been discussed.

## 2. Abiotic Stress Responses in Plants

While one or more stresses alter the plant’s most favorable environment, the plant employs a special mechanism known as “stress sensing” to detect the change. It is the extremely earliest incidence that occurs upon stressor exposure and initiates the plant stress response(s). Plants use a variety of stress sensing methods, depending on the species, organ, and type of stress [65]. For instance, light and wind stresses influence the aboveground aerial portion of the plants, while drought and salt stress affect the underground portion of the plant, triggering distinctive stress-sensing mechanisms in every case. The receptor substrate and receptor–photon binding models are the most prominent stress transmitting models for radiation and chemical stress [66]. Osmosensors in root cells can detect water availability in the soil, while the sugar production process may be used to detect stressors that alter signaling and growth [67,68].

The Mehler reaction is important in reducing the rate of photosynthetic carbon fixation during drought and salinity, as high levels of O_2_^•−^ and H_2_O_2_ are generated in chlorophyll [69]. In response to drought stress, the concentrations of ABA can rise up to 50-fold [70], which is one of the biggest increases in ABA concentration observed thus far in plants undergoing environmental stimulus. Different stresses stimulate or inhibit stomatal closure, thereby activating anion channels, along with outward K^+^ channels, and blocking inward-rectifying K^+^ channels [71,72]. During drought stress, guard cells close their stomata through a complex membrane transport system to conserve water in order to survive and maximize water use, a process that differs from transcriptional regulation for long-term drought adaptation [73]. Salinity causes plants to undergo two types of stress simultaneously, namely osmotic potential change leading to reduced water uptake and ion toxicity due to the accumulation of Na^+^ and Cl^−^ ions in the soil. Several studies have shown that the large central vacuoles of plants accumulate Na^+^ through the activity of Na^+^/H^+^ exchangers [74]. This mechanism reduces the concentration of Na^+^ in the cytoplasm by securing it within the central vacuole. In saline conditions, Na^+^ accumulated in the vacuole serves as an osmolyte and lowers the cellular water potential, thus promoting water uptake. Na^+^ is accumulated in cells with large vacuoles in roots, such as parenchyma and cortex cells, thereby reducing Na^+^ entry into root xylems [75]. In the context of cellular redox homeostasis, oxidative stress is an influential and complex phenomenon caused by an exponential enhancement in ROS [76].

Since evolving, plants have learned to cope with the changing environment by producing various enzymatic and nonenzymatic antioxidants that scavenge ROS in numerous cellular organelles in order to neutralize them [52,77,78]. The important ROS scavenging enzymes include SOD, present in almost every cellular compartment, and APX, POX, GPX, LPX, and CAT in peroxisomes [40,41,79]. The widely studied enzyme SOD, dis-mutates O_2_^•−^ into H_2_O_2_, which, subsequently, is detoxified by CAT and a broad set of POXs that further disintegrate H_2_O_2_ into water (H_2_O) and molecular oxygen (O_2_) [80]. However, these antioxidants do not counteract the uncompensated ROS accumulation during stress conditions, which leads to oxidative damage and oxidative bursts [81].

## 3. Drought Stress Responses in Plants

Water deficit or drought stress is one of the major abiotic stressors that cause dehydration and decrease crop yields due to a direct impact on all aspects of plant growth and development. The major effects of drought shock are metabolic and osmotic imbalances that result in stomatal closure and turgor loss [82]. This, in turn, prevents carbon dioxide (CO_2_) from being taken up by the cells, inhibiting cell growth and decreasing photosynthesis [83]. When a plant is stressed by a prolonged water deficit, it reaches a point of permanent wilting and dies. In general, drought stress reduces crop yields, changes chlorophyll components, hampers photosynthetic processes [84], and alters the enzyme activity that is implicated in antioxidant processes and carbon metabolism [85,86]. Under drought stress conditions, plants respond molecularly, physiologically, and biochemically, of which photosynthesis is a major physiological target [87,88]. These changes disrupt the normal homeostasis of plants. During stressful conditions, ROS such as H_2_O_2_ can cause cellular damage, toxic effects, and inhibit photosynthesis [89], whereas, in normal conditions, these molecules take part in signal transduction, enabling plant cells to maintain their normal cellular processes [79,90].

**Figure 1:**
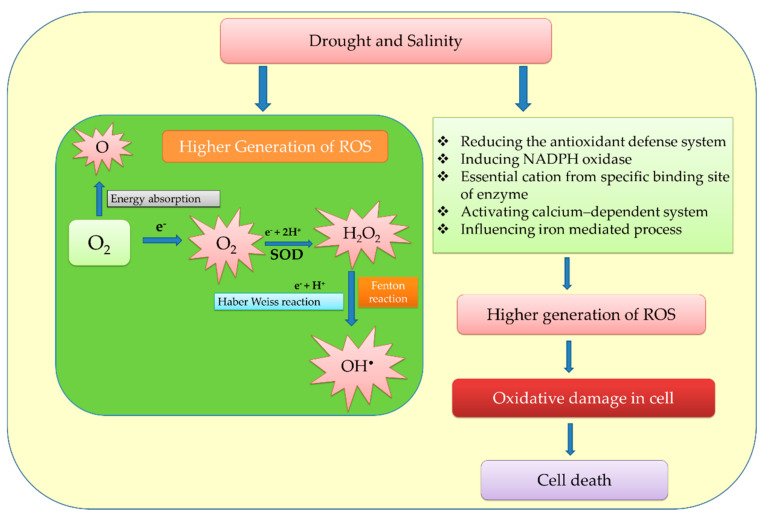
Drought and salinity-induced ROS generation in plants. Drought and salinity stress generates ROS via Fenton and Haber-Weiss reactions. ROS production by abiotic stresses modulates the enzymes (such as inducing NADPH oxidase and decreasing the antioxidant glutathione pool), activating calcium-dependent systems and altering iron-mediated processes. This led to a higher damage of ROS, thereby causing oxidative stress and damaging the cellular organelles.

Plants experience oxidative stress when water deficits exist; this leads to the production of various ROS and RNS that adversely affect plant growth, causing cellular processes to slow down [91]. In mitochondria and chloroplasts, high ROS levels cause inequities in electron transport. Photorespiration, which is the primary cause of ROS production under water deficit conditions, produces about 70% of the total H_2_O_2_. The presence of drought stress results in stomatal closure, leading to ROS accumulation [92,93]. A variety of enzymatic reactions in plants involving O_2_^•−^ and H_2_O_2_ govern diverse reactions at the cellular level, including the Fenton reaction (an iron-catalyzed reaction) and several enzyme reactions involving POXs, xanthine oxidase, lipoxygenases, and reduced NADPH oxidase (Figure 1). These radicals have the greatest tendency to damage cellular components such as carbohydrates, proteins, lipids (through the peroxidation of unsaturated fatty acids in the membrane), nucleic acid, and enzymes (through denaturation) [94].

## 4. Salinity Stress Responses in Plants

Salinity stress is a significant abiotic factor affecting agricultural systems globally. It affects more than 5% of the globe’s land area, resulting from natural processes [60,95]. It takes a long time for salt to accumulate in arid and semiarid zones [96]. Minerals and nutrients in the soil are important to plants; however, soluble salts present in excessive amounts cause ionic and osmotic stress [97]. Salinity or salt stress results from excessive accumulation of water-soluble salts like sodium nitrate (NaNO_3_), sodium chloride (NaCl), sodium sulfate (Na_2_SO_4_), potassium sulfate (K_2_SO_4_), sodium carbonates (NaHCO_3_ and Na_2_CO_3_), calcium sulfate (CaSO_4_), magnesium chloride (MgCl_2_), and magnesium sulphate (MgSO_4_) [97,98]. Almost all of these salts are essential to the plant and contribute to its growth and metabolism. Despite this, they may become toxic if overconsumed or present in excessive concentrations [97]. An optimal concentration of NaCl, for instance, maximizes plant growth, but higher concentrations completely inhibit seed germination and plant growth and development in salt-prone soils [41,99,100].

Various plants and tissues have been reported to undergo oxidative stress when exposed to high salinity [101]. Various plant traits are involved in salt tolerance, from genomic to proteomic and metabolomic levels [102]. A high level of ROS is generated in numerous plant tissues when plants are stressed by salinity due to irregularities in ETCs and the accumulation of photoreductant power. During salinity stress, proteins are altered both during transcription and post-transcriptionally [102]. Therefore, proteomics can contribute to the answers from genomics and transcriptomics. To understand the role of a protein in salinity stress tolerance, the proteomic analysis provides information not only about its down- or upregulation but also about its function, post-transcriptional modifications, and, thus, its interactions with several other proteins and its localization within the cells and tissues [103,104,105]. Different types of proteins begin to change their functional groups when exposed to salinity stress. Among these ion transporters, signal proteins and proteins participate in energy metabolism [106]. Among these proteins, annexin and calmodulin bind calcium and are activated by salinity stress [107]. Their function is to transduce ABA signals. There are several other proteins in Rab’s guanosine triphosphate-binding proteins (GTPase) family involved in transducing salinity stress signaling. Under salinity stress, the *OsRPK1* protein kinase regulates the H^+^-ATPase of the plasma membrane to restore homeostasis to the plasma membrane [108]. The presence of many of these proteins is downregulated in plants that are salt-sensitive, such as potatoes [109]. Protein degradation is another aspect of the changes observed during salinity stress. Similarly, salinity also influences lipid metabolism. The monogalactosyldiacylglycerol synthase enzyme is a component of the galactosylglycerolipids in thylakoids, and chloroplast membranes (digalactosyldiacylglycerol and monogalactosyldiacylglycerol) were reduced during salinity stress, thereby impairing the membrane integrity [107,110].

## 5. Mechanisms of ROS Regulation in Plant Stress Responses

An imbalance of various ROS species or an accumulation of ROS resulting from high glucose levels degrades active indole-3-acetic acids (IAA), precludes root growth, and impairs root meristem activity through the conserved autophagy/macro-autophagy pathway [111]. Whenever the external environment changes, the metabolic level changes, triggering the formation of ROS in plant cells [112]. Due to diverse cellular metabolic activities in higher plants, ROS production is inevitable [113]. The production of ROS was dramatically increased by different abiotic stress conditions, including metal/metalloid toxicities, drought, and salinity destroying the balance of ROS with antioxidant enzymes [114]. ROS are essential for the regulation of gene expression in an organism under normal conditions, as they regulate numerous processes inside the cell. Further, ROS regulate a wide range of functions, including the cell cycle, plant growth, signaling, abiotic stress response, programmed cell death, pathogen defense, and developmental processes [31]. During the course of photosynthesis, respiration, and other metabolic activities under stress, there is an imbalanced activation or reduction of oxygen, which results in the excessive generation of ROS in various parts of plant cells, such as peroxisomes, plastids, mitochondria, apoplasts, and cytosols that affect proteins, enzymes, chlorophylls, and ETC. The respiratory and photosynthetic ETC, plasma membrane-localized nicotinamide adenine dinucleotide phosphate (NADPH) oxidases, and apoplast POXs are major pathways that are primarily involved in ROS production in plant cells [52]. Chlorophyll is the most important component of the plant cell for ROS production [20,113,115]. ROS are produced either by retrograde signaling or by an oxidative burst in plant cells under unfavorable abiotic conditions. ROS production and detoxification are inequitable, which leads to OS that are harmful to plants. However, ROS can cause further damage unless a mechanism is activated in cellular organelles that limits their production at the beginning. Drought and salinity stress generates ROS via Fenton and Haber–Weiss reactions [116] and decreases the antioxidant glutathione pool, dislodging cations from enzyme-binding sites, altering iron-mediated processes and activating calcium-dependent systems (Figure 1) [114,117,118,119]. Furthermore, directing ROS into signaling pathways reduces oxidative harm and promotes tolerance of a single stressor or, possibly, of a group of stressors.

Oxidative stress causes ROS to be produced that can permanently damage the cellular apparatus. The major components of ROS in plants are typically formed through the excitation of oxygen to form ^1^O_2_ or the transfer of one, two, or three electrons to oxygen to create O_2_^•−^, H_2_O_2_, or OH^•^. Singlet oxygen is a highly reactive byproduct of oxygenic photosynthesis, and so, it cannot be avoided [120]. In PS II, when O_2_ reacts with chlorophyll triplet state, singlet oxygen is generated. Further, the synthesis of this ROS in both PS I and PS II is highly effective [121]. Oxidative stress propagates other species through O_2_^•−^. As an electron from the photosynthetic electron is placed on the transport chain, it reduces O_2_ in the Mehler reaction, which produces O_2_^•−^ in chloroplasts. In chloroplasts, however, the lifetime of O_2_^•−^ is dependent on CuZnSOD, which consumes O_2_^•−^ to form H_2_O_2_. Hydrogen peroxide, besides having a moderate reactivity, does not possess unpaired electrons, which means that it can move between biological membranes at a high rate, causing damage well away from its point of origin [122]. Hydrogen peroxide plays a role in many physiological processes such as seed germination, regulating stomatal apertures, and even regulating senescence and cell death [123]. OH^•^ is a form of radical with high reactivity, especially in reaction with ^3^O_2_. These ROS can damage cells by altering their lipids, proteins, and membrane conformations [124]. OH^•^ is produced at the cytosolic level by ROS-producing cell organelles like chloroplasts (especially at PS-II) or mitochondria [125]. ROS components are produced by a number of organelles, such as the nucleus, chloroplasts, plasma membranes, mitochondria (mainly in ETC), endoplasmic reticulum, and peroxisomes, which are involved especially in the photosynthetic carbon oxidation cycle [32]. The production of ROS by plants in high amounts can cause damages like protein oxidation, fatty acid oxidation or lipid peroxidation, and DNA damage. Additionally, ROS signaling is also characterized by several important components, including receptor proteins, ROS-induced inhibition of phosphatases, and redox-sensitive transcription factors [126].

## 6. Mechanism of Stress Tolerance in Plants by Modulating the Antioxidants Machinery

Plants sense stress signals through plasma membrane receptors and initiate various pathways of signal transduction from root to shoot, which employ long-distance signaling [127,128]. Initially, plant roots sense osmotic, as well as ionic, stress caused by salinity, and they alter their signaling accordingly. Several secondary messengers are involved in signal transduction, including calcium ions, ROS, inositol phosphate, and phytohormones [129,130]. In response to NaCl stress, the intracellular calcium concentration increases transiently, which activates downstream signal transduction pathways. Calcium-dependent protein kinases and calcium-binding proteins, calcineurin B-like proteins (a *SOS* family protein: *CBL4*), sense an increase in cytosolic calcium concentration in the plasma membrane, which activates ion transporters in the plasma membrane [131,132]. Tuteja and Mahajan [133] reported that Na^+^ ion transporters play a crucial role in maintaining cellular toxicity. To cope with salinity, plants change their physiology, biochemistry, and molecular mechanisms. Plants maintain their water content under osmotic stress by altering some phenotypic characteristics, such as reducing cell division and elongation, inhibiting shoot branching and lateral root formation, and closing their stomata. Furthermore, plant survival under salinity stress is dependent on the ratio of shoots to roots, because heavier roots accumulate more salts and will not allow the salts to bypass the foliage [134]. Different phytohormones regulate these phenotypic changes in plants, including cytokinin, auxin (IAA), gibberellin, ethylene, and ABA [135]. These phytohormones are interdependent and play an essential role in the integration of signaling pathways. Auxins and cytokinins play different roles in cell differentiation, division, and expansion. Through stomatal closure, ABA regulates the water potential inside a cell during osmotic stress and impacts the photosynthetic rates [136]. Abscisic acid also reduces the gibberellic acid content, thus inhibiting shoot growth and leaf expansion. During cellular processes, salt ions are confined within vacuoles, which interfere with osmotic balance. Due to this, cells become dehydrated because water oozes out of the cytoplasm into the extracellular space. In order to maintain this osmotic pressure, plant accumulates a number of low molecular weight organic compounds within their cytoplasm that are compatible with metabolism referred to as compatible solutes. Munns and Tester (2008) pointed out that oxidative stress accumulates solutes such as proline, betaine, glycine, sucrose, trehalose, and mannitol [51]. These compounds are more abundantly accumulated by halophytes (>40 mM) than glycophytes (up to 10 mM) [137]. Similarly, solutes that are compatible with membranes, proteins, and enzymes act as osmoprotectants that help in stabilizing the subcellular structure under dehydration conditions, thereby protecting the plants against oxidative damage by scavenging the free radicals.

Plants are susceptible to ionic stress under prolonged exposure to salinity, causing an accumulation of Na^+^ that causes cytotoxicity. To prevent Na^+^ toxicity, plants have developed two mechanisms: increased vacuolar sequestration or intracellular compartmentation and increased Na^+^ extrusion [138]. Plants are susceptible to salinity tolerance due to the effectiveness of these mechanisms. By excluding salts from roots, glycophytes maintain Na^+^ toxicity, whereas halophytes use a tonoplast Na^+^/H^+^ channel to improve ion compartmentation [139]. As Na^+^ accumulates in the roots, which is then stored in the xylem, it is transported to the leaves through the transpiration stream. A leaf’s tissues have a greater susceptibility to ionic stress than most other tissues. Additionally, the recirculation of Na^+^ from shoot to root leaves some amount of Na^+^ in the shoot [99,140]. Therefore, a regulatory network of different transporters is responsible for the efflux of Na^+^ from tissues and reabsorption of Na^+^ from the xylem to allow cells to tolerate ionic stress [141]. Qiu et al. [142] showed that the plasma membrane transporter Na^+^/H^+^ antiporter is responsible for the excretion of Na^+^ ions from cells. Several studies have demonstrated that Salt Overly Sensitive 1 (*SOS1*) has Na^+^/H^+^ antiporter activity in *Arabidopsis* [143,144,145,146,147]. Additionally, tonoplast antiporters facilitate ion compartmentation within vacuoles. This group includes the Na^+^/H^+^ exchangers in *Arabidopsis* [148]. Additionally, plants also inhibit Na^+^ uptake by their roots by activating *HKTs*, which have a high affinity for potassium (K^+^), making plants more tolerant to salinity [149].

Drought-tolerant plants respond to water scarcity by synthesizing osmolytes, subsequently increasing their osmotic potential [54]. Sometimes, osmolytes are also found in root exudates. Plants can generally be classified into either halophytes or glycophytes based on their potential to grow under conditions of high salinity. In some cases, halophyte plants can tolerate salinities as high as seawater or even higher [97,127]. In order to evade the destructive effects of high salinity, halophytes restrict the absorption of salt and reduce the concentration of salt in their cytoplasm and cell walls [150]. The salt-sensitive glycophytes plants cannot handle the high salinity levels and are incapable of using the approaches that the halophytes use to minimize the effects of high salinity. The cytosol, therefore, accumulates toxic concentrations of salt [97,150]. In addition to affecting plant roots and tubers, salinity also affects seedling viability and seed germination [41,65,127]. In extreme cases, ionic and osmotic stress causes the roots to lose their water uptake, which leads to reduced growth and metabolism [97,98]. A prolonged salinity stress causes stomata to close, which reduces the CO_2_ uptake. The reduced photosynthetic capacity caused by salt toxicity ultimately results in senescence and death, because the plant is unable to maintain an appropriate growth rate [82,97,98].

**Figure 2:**
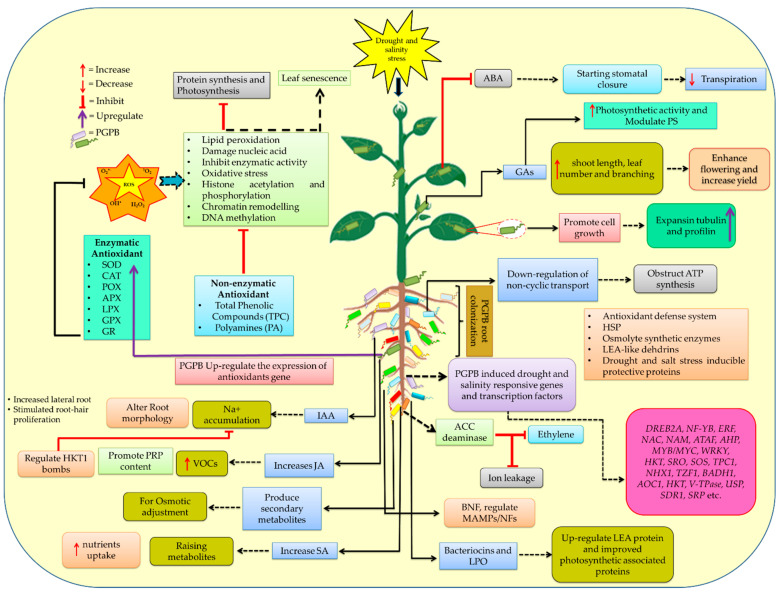
Schematic representation of plant growth-promoting bacteria (PGPB)—mediated drought and salinity stress tolerance in plants. During drought stress, the plant itself and PGPB are able to detoxify ROS into stable nonreactive compounds. SOD—superoxide dismutase; CAT—catalase; GR—glutathione reductase; GPX—glutathione peroxidase; MAMPs—microbe-associated molecular patterns; NFs—nodulation factors. PGPB modulates the signaling pathways involved in drought and salt response biochemically and molecularly. Drought response is primarily regulated by ABA, which controls other signaling pathways such as SA, IAA, JA, and GA. SA—salicylic acid; ABA—abscisic acid; JAs—jasmonic acid; GAs—gibberellins; IAA—indole-3-acetic acid. PGPR also modulates transcription factors (TFs) that are essential in the drought and salt response and tolerance. *NAM, ATAF, NAC, MYB/MYC,* and *WRKY*—transcription factors; *NF-Y*—nuclear factor-Y; *ERF*—ethylene-responsive element-binding factor; *LCOs*—Lipo-chitooligosaccharides; *BNF*—Biological Nitrogen Fixation; *AHP*—cytokinin-related genes; *AOC1*—allene oxide cyclase; *HKT*—High-affinity K^+^ transporters; *NHX1*—vacuolar Na^+^/H^+^ antiporter gene; *BADH1*—Betaine aldehyde dehydrogenase 1; V-*ATPase*—Vacuolar-H^+^-pyrophosphatase; *USP*—Cytosolic universal stress protein; *SDR1*—salt and drought-responsive gene; *LEA*—late embryogenesis abundant; *TIP1*—Tonoplast AQP gene; *SRP*—Salt-responsive protein-encoding gene; *SOS1*—Salt overly sensitive gene. Figure created with BioRender.com (https://app.biorender.com/biorender-templates)—accessed on 27 January 2022.

Additionally, plant cells also possess mechanisms for reducing ROS-induced toxicity. To combat higher levels of ROS, plant cells produce secondary metabolites such as tocopherols; phenols; flavonoids; carotenoids; polyamines; phenolic compounds; and antioxidative enzymes like SOD, CAT, POX, APX, LPX, and GSH reductase, which help in the deactivation of active oxygen species during multiple redox reactions, making the antioxidant system protective against oxidative stress [40,41,64,151]. These ROS scavengers may improve plants’ tolerance to drought and salinity stress (Figure 2). It is one of the key enzymes in plants’ defense systems against oxidative stress, as it appears ubiquitously in all of the cells of all kinds of plants. These antioxidants combat ROS by converting them into nonreactive forms. To reduce the amount of ROS in the cell, a number of antioxidative enzymes cooperate within different cellular compartments [152]. Under drought and salinity stress conditions, plants show enhanced antioxidant enzyme activities as a mechanism of tolerance to stress [153]. Studies have reported that *Arabidopsis* overexpresses aldehyde dehydrogenase and is resistant to drought and salinity [154,155,156]. By overexpressing *Chlamydomonas* GPX in chloroplasts or cytosols of transgenic tobacco plants, stress tolerance can be improved [157].

## 7. PGPB for Plant Growth Promotion and Stress Tolerance

Plants, unlike many other organisms, have evolved different mechanisms to guard themselves against stressful conditions and promote growth and development, as well as to avoid, and protect themselves from, stressful conditions [17,158]. The application of useful bacteria to increase drought and salt tolerance in plants is a substitute that is cheaper and more feasible [50,159,160,161]. Numerous studies have revealed that PGPB can improve both plant growth and nutrition of a variety of crops even when adverse environmental conditions occur, including drought and salinity [18,41,58,63,162,163,164,165,166]. PGPB affects plants directly by producing phytohormones or indirectly by inducing signaling in the host. Most commonly, phytohormones like IAA, gibberellins, cytokinin, ABA, and ethylene; biological nitrogen fixation (BNF); and phosphate solubilization are attributed a direct role [41,63,64]. However, the indirect mechanisms include the production of hydrogen cyanide, antibiotics, volatile organic compounds (VOC), siderophores, and ammonia that suppress phytopathogens. In addition to improving crop water relations and changing the ion balance, these soil microorganisms also modulate abiotic stress regulation via different pathways [50,160]. Over the last several decades, PGPB has been broadly used for sustainable agriculture in several parts of the world in order to reduce chemical pesticides and fertilizers [63,167,168].

### 7.1. Role of PGPB on Drought Stress Tolerance

**Figure 3:**
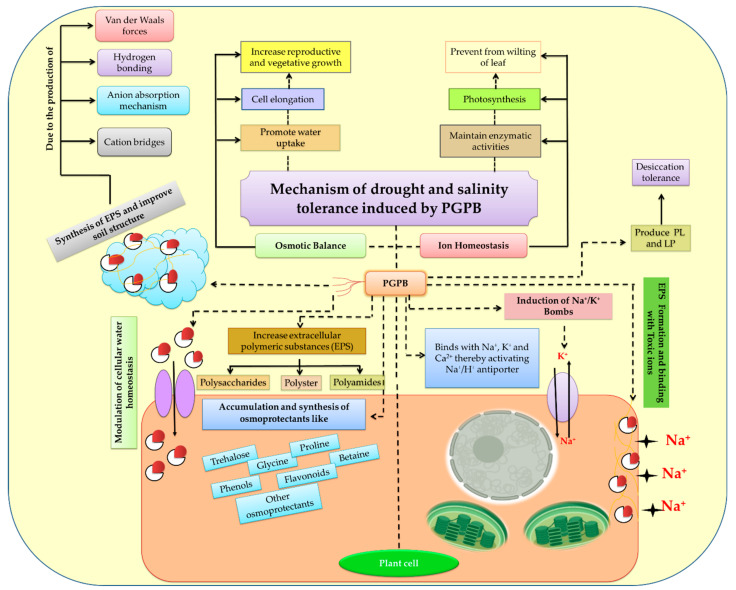
Mechanisms of plant growth-promoting bacteria (PGPB)-induced tolerance of drought and salinity stress. Plant inoculated with PGPB experienced growth-promoting attributes like EPS and ESP production that modulate cellular water homeostasis. PGPB also induces the accumulation and synthesis of various osmoprotectants like trehalose, proline, glycine, phenols, flavonoids, and so on that help in scavenging ROS and RNS in cells. PGPB are also responsible for maintaining the ion homeostasis (Na^+^/K^+^) and removing the toxic ions from the cell. EPS—extracellular polymeric substances; ESP—exopolysaccharide; PL—polysaccharide lipid; LP—lipopolysaccharide protein; Na^+^—sodium ion; K^+^—potassium ion. Figure created with BioRender.com (https://app.biorender.com/biorender-templates)—accessed on 21 November 2021.

Numerous PGPBs synthesize osmolytes and help the plants cope with drought stress (Figure 3). It has been suggested that the production of IAA by PGPB may contribute to the increase in root–shoot biomass under drought stress [169]. Plants are also known to regulate their growth with ethylene, whose production is influenced by conditions such as drought, salinity, and waterlogging [170]. A rhizospheric bacteria producing aminocyclopropane-1-carboxylate deaminase (ACCD) inhibits the ethylene signaling pathway to resist root drying. In tomato and pepper plants, *Achromobacter piechaudii* exhibits ACCD activity, leading to an improvement in biomass by resisting the water deficit. Similar results were also reported by references [40,41] under salinity stress on pea plants. ACCD-positive isolates reduce the overproduction of ethylene in plants, which enhances injuries caused by water scarcity without affecting the relative water content (RWC) of plants [171]. Plants that have been injected with drought-tolerant bacteria achieve better plant growth and have higher proline contents in their roots and leaves. The effect of PGPB is significant in the presence of water [172]. According to Creus et al. [173], the inoculation of *Azospirillum* under water scarceness reduced the yield of wheat and increased the ion contents, such as magnesium (Mg^2+^), potassium (K^+^), and calcium (Ca^2+^), in grains. Researchers have found that PGPBs, together with plant growth regulators, provide tolerance to plants under drought stress [174,175,176]. Plant growth and development are significantly influenced by PGPB. In addition to providing micronutrients to the host plants, they can also enhance the availability of growth-promoting chemicals. For instance, they produce exopolysaccharides (ESP), a type of carbohydrate that is released in the rhizospheric region [177]. These ESPs perform a vital function in protecting plants from desiccation [178]. Salicylic acid (SA), a well-known phenolic compound that is secreted by microorganisms, is required for plant growth and development, thereby providing drought tolerance (Table 1 and Figure 3). It works as a signaling molecule under drought stress, triggering genes that function as heat shock proteins (HSP), chaperones, antioxidants, and activate genes that synthesize secondary metabolites [179,180].

#### 7.1.1. Metabolic Reprogramming

Plants under drought stress may be reprogrammed by numerous microbes by regulating their metabolism and molecular pathways. Some metabolites such as pyruvic acid (PA), succinic acid, thiamine pyrophosphate, uridine diphosphate, and dihydroxyacetone are significantly decreased in wheat under drought conditions, whereas *Bacillus velezensis* treatment helps to make these metabolites more available to combat drought stress [181]. *Azotobacter brasilense, A. chroococcum*, and *Bacillus* sp. increase the accumulation of soluble sugars, proteins, phenols, flavonoids, ABA, and oxygenated monoterpenes in pennyroyal during drought and salinity stress conditions [40,41,182]. Furthermore, a consortium of bacteria, including *B. thuringiensis, B. subtilis*, and *B. megaterium*, significantly increased the levels of glycerol, L-asparagine, nicotinamide, riboflavin, total sugar, and 3-hydroxy-3-methylglutarate in chickpea leaves under drought stress [183]. Consequently, plant-associated bacteria may be able to alter the metabolic changes induced by drought in order to reduce the effects.

**Table 1:** PGPB-produced mechanisms related to tolerance against drought stress.

PGPB	Plants	Effects	Mode of Action	References
*Azospirillum brasilense* Sp245	*Triticum aestivum*	A higher Mg^2+^, K^+^, and Ca^2+^ content in the grain, as well as higher water content, RWC, and water potential	N_2_ fixation	[173]
ACCD producing rhizobacteria	*T. aestivum*	Increased root-shoot length, biomass and lateral root number	ACCD production	[184]
*A. piechaudii*	*Lycopersicon esculentum*	A remarkable mechanism of stress resistance has been found in the production and excretion of glucosyl glycerol.	Use transcriptomic and microscopic approaches to assess osmotic stress tolerance	[185]
*A. xylosoxidans* Cm4, *Variovorax paradoxus* 5C-2 and *Pseudomonas oryzihabitans* Ep4	*Solanum tuberosum*	Increased plant biomass	Decrease amino acid and ethylene content	[186]
*Acinetobacter calcoaceticus* WP19, *Rahnella* sp. WP5, *Burkholderia* sp. WP9, *Enterobacter asburiae* PDN3, *Pseudomonas* sp. WW6, *Sphingomonas yanoikuyae* WW5and *Curtobacterium* sp. WW7	Poplar/*Populus*	Plant growth promotion (increased root-shoot dry weight, total dry weight, total nitrogen); enhanced protection against ROS	Reduced ROS damage, phytohormone production and microbial genes identification for drought tolerance	[187]
*Azospirillum* sp.	*T. aestivum*	Increased lateral roots formation and root growth, and uptake of nutrients and water content	Production of IAA, high amount of nitrogen, P-solubilization and ACCD activity	[188]
*B. megaterium* CAM12 and *P. agglomerans* CAH6	*Vigna radiata*	Reduced Aluminium uptake in plants; increased plant biomass (Plant growth promotion); higher content of chlorophyll and carotenoids	Increase IAA production, ACCD activity, EPS and ESP production, siderophore production	[189]
*B. thuringiensis*	*Lavandula dentate*	Modulate antioxidants enzymes like APX and GR	By controlling shoot proline accumulation and depressing stomatal conductance, IAA increased K^+^ content	[190]
*B. cereus* AR156, *B. subtilis* SM21, and *Serratia* sp. XY21 (BBS)	*Cucumis sativus*	Proline content of the leaves was increased; enhanced SOD activity in a significant way	BBS treatment downregulate the expression of *rbcL*, *cAPX*, and *rbcS* genes	[191]
*B. megaterium* and *Glomus* sp.	*Trifolium*	Increase antioxidant enzymes like GR, SOD, and CAT	IAA and proline production	[192]
*B. megaterium* BOFC15	*Arabidopsis thaliana*	Improved root system architecture, enlarged plant biomass, and increased photosynthetic capacity	Elevates cellular polyamine (spermine, spermidine), isoprenoid, ABA, and reduces malonaldehyde content	[193]
*B. polymyxa*	*L. esculentum*	Physiological and biochemical characteristics of plants were improved by proline accumulation	Phosphate solubilization	[194]
*Bacillus* sp. KB142, KB133, KB129 and KB122	*Sorghum bicolor*	Increased plant biomass, RWC, chlorophyll content and soil moisture content	Increase ESP production and Biofilm formation; accumulation of proline and sugars;	[195]
*B. subtilis* GB03	*A. thaliana*	Expression of the PEAMT gene in osmotically stressed plants improved leaf RWC and dry DMW as well as the metabolic level of glycine betaine and choline.	Enhances the biosynthesis of Cho and Gly Bet in *Arabidopsis*; increases ABA synthesis	[196]
*B. thuringiensis* AZP2	*T. aestivum*	Increasing photosynthesis and reducing volatile emissions	ACCD production and P-solubilization	[197]
*B. polymyxa*	*L. esculentum*	Increased RWC, protein, chlorophyll, proline accumulation and yield	Phosphate solubilization	[194]
*B. phytofirmans*	*T. aestivum*	Improved water-use efficiency, photosynthetic rate, chlorophylls content, nitrogen (N), phosphorus (P), potassium (K), and protein levels in wheat grains	Ameliorating the RWC, improving chlorophyll content and photosynthetic rate	[198]
Consortia containing *P. synxantha,* R81 *P.jessenii,* R62, and *Arthobacter nitroguajacolicus* strainYB3, strain YB5	*Oryza sativa*	Accumulation of proline improved plant growth and osmotic adjustment	PGPR increases the proline content, CAT, SOD, APX, POX, LPX, and lower level of H_2_O_2_, content	[199]
Consortia of *Bacillus* isolate 23-B and *Pseudomonas* 6- P with *Mesorhizobium ciceris*	*Cicer arietinum*	Higher proline concentration, improved seed germination, root-shoot length and fresh weight of the seedlings	ACCD production	[200]
*E. mori* AL, *E. asburiae* BL and *E. ludwigii* CL2	*T. aestivum*	Increased plant biomass	Higher ACCD production	[201]
*Gluconacetobacter diazotrophicus* PAL5	*Saccharum officinarum*	Drought resistance is conferred by the activation of ABA-dependent signaling genes	Activate drought-responsive markers and hormone pathways, such as ABA and Ethylene.	[202]
*Ochrobactrum pseudogrignonense* RJ12, *Pseudomonas* sp. RJ15, and *B. subtilis* RJ46	*V. mungo* and *Pisum sativum*	Plant growth promotion (enhanced seed germination, percentage, root-shoot length, and dry weight), enhanced cellular osmolytes and ROS scavenging enzymes, enhanced leaf chlorophyll content	ACCD production	[203]
*Paenibacilluspolymyxa* and *Rhizobium tropici*	*Phaseolus vulgaris*	Increased plant growth, N_2_ content, and nodulation	ACCD production	[204]
*P. putida* H-2–3	*Glycine max*	Gibberellins secretion improved plant growth, induced regulation of stress hormones and antioxidants and also increased the crop productivity	Gibberellin production and increased antioxidants enzymes	[205,206]
*P. polymyxa* B2	*A. thaliana*	Induction of *EARLY RESPONSE TO DEHYDRATION 15* (*ERD15*)	Produce antibiotic compounds, Hydrogen cyanide and Siderophore	[207]
*Phyllobacterium brassicacearum* STM196	*A. thaliana*	Increased plant biomass, lowers transpiration and photosynthesis	Modulate ABA content, delayed reproductive timing	[208]
*P. brassicacearum* strain STM196	*A. thaliana*	Reduced leaf transpiration was caused by increased ABA content	Modulate ABA content, delayed reproductive timing	[208]
*P. brassicacearum*	*A. thaliana*	Increased biomass, ABA content, higher water-use efficiency	Confer stress tolerance by modulating the biochemical parameters	[208]
*P. chlororaphis* O6	*A. thaliana*	Transcripts of the jasmonic acid marker genes, *pdf*-1.2 and *VSP1*, ethylene-response gene, *HEL, PR-1* and SA-regulated gene were up-regulated in colonized plants	Response to ROS, and auxin- and jasmonic acid-responsive genes	[209]
*P. fluorescens* biotype G (ACC5)	*P. sativum*	Induced longer roots and water uptake	ACCD production	[210]
*P. putida*	*C. arietinum*	Osmolyte accumulation (proline, betaine, glycine) and ROS scavenging	IAA production and ACCD activity	[211]
*P. putida* P45	*Helianthus annuus*	An increase in rhizosphere nutrient and water uptake	ESP production	[212]
*Pseudomonas* sp.	*P. sativum*	Decreased ethylene production	ACCD production	[204]
*P. aeruginosa*	*V. radiata*	Increased root and shot length, dry weight and RWC	Production of ROS scavenging enzymes and up-regulation of three drought stress responsive genes (*CAT*, *DREB,* and *DHN*)	[213]
*P. putida*	*H. annuus*	Increased plant biomass, biofilm formation on roots and soil adhesion	ESP production	[212]
*R. etli* overexpressing trehalose-6-phosphate synthase gene	*P. vulgaris*	Signaling molecules like trehalose upregulate genes involved in carbon metabolism, nitrogen metabolism, and stress tolerance	Increased activity of nitrogenase gene and overexpression of trehalose-6-phosphate synthase	[214]
*R. phaseoli* (MR-2), *R. leguminosarum* (LR-30), and *M. ciceri* (CR-30 and CR-39)	*T. aestivum*	IAA produced by the consortia improved biomass, growth and drought tolerance index	ESP production and increased catalase activity	[215]
*V. paradoxus* 5C-2	*P. sativum*	Growth, yield, nodulation, production and water use efficiency are increased with xylem abscisic acid	Induced ABA and ACCD production	[216]
*Consortia of B. amylolequefaciens and P. putida*	*C. arietinum*	Growth, production and drought stress tolerance	IAA production, ACCD activity, P solubilization, Siderophore activity	[64]

RWC—Relative Water Content; N_2_—Nitrogen; ACCD—1-aminocyclopropane-1-carboxylate deaminase; APX—Ascorbate peroxidase; GR-Glutathione reductase; ROS—Reactive Oxygen Species; Cho—Choline; GlyBet—Glycine Betaine; IAA—Indole Acetic Acid; EPS—Extracellular Polymeric Substances; ESP—Exopolysaccharide; K^+^—Potassium; SOD—Superoxide Dismutase; CAT—Catalase; POXs—Peroxidases; LPX—Lipid Peroxidase; H_2_O_2_—Hydrogen Peroxidase; ABA–Abscisic Acid; SA—Salicylic acid; DREB—Dehydration-Responsive Element-Binding Protein; *DHN*—Dehydrin; VSP1—Vegetative storage protein; HEL—Ethylene responsive gene; PR-1—SA-regulated gene.

#### 7.1.2. Biochemical Changes and Molecular Adaptations

Plants respond to drought and salt stress through a variety of processes, including the production of distinct proteins, the secretion of metabolites, and the modulation of genetic expression. Several PGPBs play an essential role in the production of numerous phytohormones (such as IAA, GAs, cytokinin, etc.). These phytohormones play a critical role in regulating plants’ growth and response during salinity stress and water deficit conditions. When the plants are exposed to drought and salinity stress, these phytohormones stimulate various signaling pathways, which result in the greater production of secondary metabolites, antioxidant enzymes, and HSP. Hence, the study and development of numerous phytohormone-related strategies is necessary for increasing drought and salinity tolerance in plants [217].

Apart from biochemical processes, PGPB also modulates molecular mechanisms such as inducing the production of lipochitooligosaccharides (LCOs), late embryogenesis abundant (LEA) proteins, regulating microbe-associated molecular patterns (MAMP), and nodulation factors (NFs), as well as activating several drought and salt-responsive genes (Figure 2). Cellular dehydration tolerance is mediated by LEA proteins [218]. Several microbes produce LCOs, which trigger symbiotic interactions with plants [219]. In response to the flavonoids in root exudates, rhizobacteria secrete Nod factors (NFs), which induce nodule formation [218,220]. Plants have high-affinity K^+^ transporters (HKT) located on their plasma membranes that mediate Na^+^ transport, preventing Na^+^ ions from building up during photosynthesis by excluding them from the shoots (Figure 2) [40,221,222]. Salt stress also ensures cell integrity by increasing the levels of tubulin and profilin, which bind to actin and regulate the cytoskeleton structure [223,224,225,226]. In drought stress, dehydration-responsive element-binding protein 1 (*DREB1*)/CBF (C-repeat binding factor) and DREB2 regulons function to control the gene expression in an ABA-independent manner [227]. Allene oxide cyclase (*AOC1*), an enzyme involved in the α-linolenic acid metabolism pathway, was found to increase the salt tolerance in both wheat and *Arabidopsis* [228]. Here, we outlined and discussed the numerous molecular and biochemical response mechanisms in Figure 2. There are several biocontrol agents and plant growth-promoting substances that are produced by rhizospheric microbes that live around plants [229]. Moreover, they increase the nutrient availability and influence the soil structure, pH, fertility, and oxygen availability [230]. Through numerous processes within the rhizosphere and phyllosphere, these microbes increase the ability of plants to tolerate drought and salinity, thereby promoting plant growth (Figure 2).

### 7.2. Role of PGPB on Salinity Stress Tolerance

Salinity affects plants in two main ways. High salinity makes the soil hard and dry, which hinders roots from extracting water and causing toxicity to plant cells, thereby affecting plant growth and metabolism. However, toxic concentrations of salt take longer to accumulate in plants [51]. PGPB can alleviate the severity of salinity-related problems. It has been reported that Gram-positive (G^+^) and Gram-negative (G^−^) PGPB can colonize the roots of plants and decrease the effects of salinity through direct and indirect mechanisms [62,231,232] (Table 1 and Table 2). These bacteria exhibit chemotaxis and produce ESP, IAA, and ACCD that can withstand salinity stress (Figure 3) [40,41,233]. With PGPB, plants can develop induced systemic tolerance that enables them to cope with the salinity stress [234]. In a study by Yildirim et al. [235], they found that *Staphylococcus kloosii* and *Kocuria erythromyxa* can induce salinity tolerance in *Raphanus sativus* by producing an antioxidants enzyme that scavenges ROS [235,236]. Another study by Nadeem et al. [237] demonstrated that *P. fluorescens, P. syringae*, and *E. aerogenes*, possessing ACCD activity, could induce salinity tolerance in maize by regulating the K^+^/Na^+^ ratios, chlorophyll levels, and proline levels. According to Hamdia et al. [238], the inoculation of *Azospirillum* with a high K^+^/Na^+^ ratio improved the salt tolerance in maize. According to M’Piga et al. [239], PGPB acts against a variety of phytopathogens by inducing numerous defense enzymes like phenylalanine ammonia-lyase (PAL), POX, chitinase, and β-1,3-glucanase (GLU). It has been demonstrated that IAA acts on the H^+^ ATPase of the plasma membrane, thereby causing Na^+^ ions to load into root cells [144,240]. Under salinity stress, another strain of *Pseudomonas* sp. PDMZnCd2003 can produce high concentrations of IAA. The molecular study reveals that plants suffering from salinity stress had higher levels of ethylene due to higher levels of ACC, which causes changes in various physiological functions. Plants may grow better by any mechanism that reduces the ethylene levels during salinity stress. Under salinity stress conditions, PGPB produced a variety of phytohormones that are capable of enhancing the leaf area, root growth, and a number of root tips, resulting in enhanced nutrient uptake (Table 2) [241]. It has also been demonstrated that *P. extremorientalis*, *P. chlororaphis, P. putida,* and *P. aurantiaca* can produce IAA in a 4% NaCl solution, thereby increasing the plant biomass of *Sulla carnosa* under salinity stress [241].

#### 7.2.1. Production of Extracellular Polymeric Substances

Many biopolymers were synthesized by microorganisms under natural conditions, including polysaccharides, polyesters, and polyamides (Figure 3). A variety of multifunctional polysaccharides are produced, including intracellular, extracellular, or ESP, structurally [242,243,244]. EPS-producing PGPBs are capable of alleviating salinity stress [245,246], as they bind with cations, such as Na^+^, and decrease plant accessibility towards these toxic ions. For the classification of stress-tolerant microbes, EPS may be an important criterion, as they help crops thrive under stressful conditions. EPS enhances the water retention capacity of bacteria and regulates the diffusion of organic carbon sources to promote their survival. The desiccation tolerance of bacteria is also attributed to polysaccharide–lipid (PL) and high molecular weight lipopolysaccharide–protein (LP), a carbohydrate complex. Moreover, bacteria also contain polysaccharide–lipid complexes (PLs) and high molecular weight lipopolysaccharide–protein complexes (carbohydrate complexes) that are primarily responsible for desiccation resistance. In addition, EPS can facilitate microbe–plant interactions [244,246,247] by providing microenvironments that enable microbes to survive in stressful conditions. In addition, it helps bacteria colonize the plant by allowing them to attach to root exudates. Under drought and salinity stress conditions, the EPS composition and concentration dramatically change. Microbes secrete EPS in the form of slime material in soil, which is bonded to soil by hydrogen bonds, cation bridges, anion adsorption mechanisms, etc. [248,249]. Thus, slime substance forms around soil aggregates, providing protection against drought and salinity. Plants that are inoculated with EPS-producing microbes display resistance against water deficit and salination conditions (Figure 3). By producing EPS around roots, soil microbes can also increase the water potential and increase the nutrient uptake by plants [246,249]. Under drought and salinity stress, this formation of biofilms is a common mechanism by which various microbes protect themselves from adverse effects. EPS plays an essential role in providing structural stability to biofilms [250,251]. Under saline conditions, EPS may modulate the chemical and physical characteristics of microbes and restrict sodium (Na^+^) uptake [246].

#### 7.2.2. Osmotic Adjustment

Among the effects caused by salinity, the first is osmotic stress, which disrupts the water balance, resulting in stomatal closure [60,252]. The reduced leaf area and imbalanced gas exchange lead to a decrease in the photosynthesis rates [50,253]. In addition, there is photosynthetic feedback inhibition. During reduced growth, carbohydrates accumulate in storage organs and meristems, which are otherwise used in the expansion and proliferation of new tissues [160,253]. In order to compensate for the effects of drought and salinity, plants must maintain their water balance and preserve their photosynthetic structures. Through various mechanisms, PGPB has demonstrated potential application as a method of enhancing the osmotic balance (Figure 3 and Table 1).

Microbiota (especially, PGPB) are defending themselves against stressful environmental conditions (such as temperature, drought, salinity, and pH) and adhering to biotic and abiotic surfaces with the help of extracellular polysaccharides (EPS) or ESP [159,254,255]. Based on the strain and conditions, the ESP composition and amount can vary [256]. In addition to plant–microbe interactions, ESPs have additional functions. Polysaccharides increase soil particle adhesion and promote macropore generation, which increases the soil porosity and aeration (Figure 3) [159,160,257,258]. In this way, soil particles are bound, and the structure of the soil is improved, thus reducing the effects of the initial osmotic stress [60]. According to one study, when *P. mendocina* (PGPB) and *Glomusintra radices* (arbuscular mycorrhizal fungus) were co-inoculated in lettuce, they produced ESP, which produced a high percentage of stable aggregates in soil under field conditions [259]. Furthermore, Qurashi and Sabri (2012) [260] reported that both chickpea growth and soil structure were improved through the inoculation of two bacterial strains, *Planococcus rifietoensis* RT4 and *Halomonas variabilis* HT1, in *C. arietinum* plants that are subjected to soil aggregate formation under salt stress conditions [260].

Salinity reduces the growth of unused photosynthates and is a feedback inhibitor of growth. During the salinity osmotic phase, microorganisms regulate the source–sink relationship of soluble sugars in plants to favor osmotic adjustment and avoid photoinhibition feedback (Figure 3) [50,261]. The roots of plants are a strong source of carbohydrates, and their development can be influenced by the hormonal responses (IAA) associated with the actions of microbes on them. Additionally, microbes can consume a substantial portion of these photosynthates; for instance, *Medicago ciliaris* lines exhibited salt resistance through the maintenance of nodular symbiotic and sink–source activities [262]. In addition, inoculation with numerous *Bacillus* strains in wheat and strawberry has increased a variety of physiological parameters, like stomatal conductance or productivity or photosynthesis [263], and nutritional content [264]. Plants can lose intracellular water when subjected to saline stress [265]. To maintain their osmotic state of the cytoplasm and to improve plants’ responses to such stress, vegetative species produce organic osmolytes in the cytoplasm [256]. Apart from that, beneficial bacteria like *Azospirillum* [266], *Burkholderia* [222,267], *Arthrobacter* [268], *Bacillus* [268,269], *Pseudomonas*, and *Rhizobium* [58] also led to the production of certain osmoprotectants, and among them are proline, betaine, trehalose, glycine, phenols, and flavonoids (Figure 3) [160,253,265]. These mechanisms are also used by salt-tolerant bacteria to cope with fluctuating osmotic conditions [256]. Furthermore, osmoprotectants produced in bacteria are biosynthesized more rapidly than in their associated plants [253]. Studies have shown that PGPB inoculation increases plants’ osmolytes levels. The improvement may be due to bacterial solutes being absorbed by roots, or PGPB may enable the de novo synthesis in plants [160,253]. Furthermore, it was demonstrated that the usage of numerous bacterial isolates (*B. tequilensis* MPP8, *B*. *megaterium* MPP7, *P. putida* MPP18, *Alcaligenes faecalis* IG27, *A. bereziniae* IG2, and *E. ludwigii* IG10) increased the amount of TSS and proline in salt-stressed wheat plants, thereby reducing the electrolyte leakage, reducing the oxidative damage, and enhancing the amount of ROS scavenged [270,271]. Furthermore, mutants of the gene encoding trehalose synthase (*treS*) in *Pseudomonas* sp. have been constructed, and their roles in protecting plants against salinity stress have been reported [272]. A high-salt concentration alters the plant’s water potential; therefore, PGPB improves the hydraulic conductivity, thereby regulating the water homeostasis (Figure 3) [160,253,273]. A positive regulation of plasma membrane intrinsic protein (PIP)-type plasma membrane aquaporins was found after exposure to *B. megaterium* B26 in maize plants under salinity (2.59 dS m^−1^) [274]. Under the salinity stress conditions (200-mM NaCl), *A. brasilense* AZ39 inoculation also improved the transcription of a PIP-type aquaporin in barley plants [275].

**Table 2:** PGPB-produced mechanisms related to tolerance against salinity stress.

PGPB	Plants	Effects	Mode of Action	References
*P. mendocina*	*Lactuca sativa* L.	Stable soil aggregates in high proportions	ESP production	[259]
*A. brasilense* and *Pantoea dispersa*	*Capsicum annuum* L.	Increased dry weight and K^+^/Na^+^ ratio	Maintaining of higher stomatal conductance	[276]
*B. aquimaris*	*T. aestivum* L.	Increased weight, biomass, and leaf nutrients	Accumulation of osmoprotectants (TSS and proline)	[264]
*Rhizobium* sp. and *Pseudomonas* sp.	*Zea mays* L.	Increased plant biomass, development, and nutrient uptake	Accumulation of osmoprotectants (proline, Betaine), water and ion homeostasis	[58]
*Pseudomonas* sp.	*S. lycopersicum* L.	Higher shoot and root length, total dry weight, and chlorophyll content	ACCD production and osmoprotectants accumulation (trehalose)	[272]
*B. megaterium*	*Z. mays* L.	Higher root hydraulic conductance	Up-regulation of aquoporin genes (PIPtype)	[274]
*B. subtilis*	*Puccinellia tenuiflora* SCRIBN. & MERR.	Improved shoot and root growth and decreased Na^+^ ion accumulation	Ion transport genes (HKT type): transcriptional changes	[277]
*P. simiae*	*G. max* L.	Higher weight, length, and K^+^/Na^+^ ratio	Changes in transcriptional regulation of phosphatase activity, proline accumulation, and the production of VOCs	[278]
*Rhizobium* sp. and *Pseudomonas* sp.	*Z. mays* L.	Enhanced plant biomass, nutrient uptake and development	Accumulation of proline, water and ion homeostasis	[58]
*Pseudomonas* sp. and *Bacillus* sp.	*G. max* L.	Increased water content, plant biomass, and photosynthetic activity	Production of IAA ESP, and ACCD and accumulation of proline	[279]
*B. aquimaris*	*T. aestivum* L.	Increased weight, biomass, and leaf nutrients	Accumulation of osmoprotectans (PRP and TSS)	[264]
*A. lipoferum*	*T. aestivum* L.	Enhanced plant weight and chlorophyll content	N_2_ fixation and IAA production	[280]
*Bacillus* sp.	*P. sativum L.*	Enhanced morphological and biochemical parameters	IAA production, P-solubilization, ACCD, and hydrogen cyanide production	[41]
*Bacillus and Pseudomonas* sp.	*P. sativum* L.	Enhanced morphological and biochemical parameters and modulated antioxidant genes	ACCD production	[40]
*A. piechaudii*	*S. lycopersicum* L.	Increased dry and fresh weight, and K and P uptake	ACCD production	[171]
*Burkholdera cepacian*, *Promicromonospora* sp. and *A. calcoaceticus*	*C. sativus* L.	Enhanced biomass, photosynthetic pigments, water, and P and K content	Downregulation of ABA genes	[205]
*Kocuria rhizophila*	*Z. mays* L.	Reduction of Na^+^ accumulation and increase in productivity parameters	Transcriptional changes in ion transporter genes (NHX and HKT-type) and hormonal changes (ABA and IAA)	[281]
*B. amyloliquefaciens*	*Menthax piperita* L.	Improved morphological characteristics and higher chlorophyll content	VOCs production and reduction of ABA endogenous levels	[282]
*Bradyrhizobium japonicum* and *B. thuringiensis*	*G. max* L.	Germination of seeds and proteome changes	Lipo-chitooligosaccharide and bacteriocin production	[218]

ESP—Exopolysaccharide; VOCs—Volatile Organic Compounds; ACCD—1-aminocyclopropane-1-carboxylate deaminase; ABA—Abscisic acid; IAA—Indole acetic acid; PRP—Proline-rich protein; TSS—Total soluble sugar; P—Phosphorus; N_2_—Nitrogen; NHX—vacuolar Na^+^/H^+^ antiporter; HKT—Sodium transporter.

#### 7.2.3. Ion Homeostasis

Salts cause the accumulation of numerous ions such as Na^+^, Cl^−^, Ca^2+^, Mg^2+^, SO_3_^2−^, or CO_3_^2−^, which leads to ion toxicity. The influx of these ions is greater than the rate of exclusion when exposed to high salt concentrations for a prolonged period of time [51,253]. Initially, plants compartmentalize excess salts in their vacuoles to avoid accumulation in the cytosol and intracellular spaces [50,253], which would limit photosynthesis and respiration. In biochemical reactions, Na^+^ replaces K^+^, which results in protein synthesis and conformational changes [60,221]. Researchers have reported that soil microorganisms maintain ion homeostasis, which is necessary for plant development and tolerance during salinity stress [160,283]. Conversely, the majority of these studies have only considered NaCl-induced salinity stress, not the other ions involved in salinity stress. By maintaining high K^+^/Na^+^ ions ratios, PGPB can control toxic ion homeostasis, preventing the accumulation of Na^+^ and Cl^−^ ions in the leaves, increasing ion exclusion by roots, or modulating ion transporter expression [160,253,284]. The high-affinity K^+^ transporter (*HKT*) is a plasma membrane protein that mediates Na^+^ ion transport in plants, preventing the overaccumulation of Na^+^ ions in shoots by excluding excess Na^+^ ions in the roots [221,222]. Rhizobacteria have been shown to modulate the expression of these transporters during inoculation. Furthermore, by inoculating *Zea mays* L. with *K. rhizophila* Y1, the expression of ion affinity transporters (*ZmNHX3, ZMNHX2*, *ZmNHX1*, and *ZmHKT1*) was upregulated, providing protection from salinity stress [281]. Thus, plant–microbe interactions require the tissue-specific regulation of HKT-type genes to maintain ion homeostasis during salt stress [221,222,277]. In addition, another enzyme is capable of acting as a sodium antiporter, the Salt Overlay Sensitive (*SOS*) gene, which can help plants cope with salinity stress [270]. The inoculation of wheat plants with three bacterial strains (*B. tequilensis* MPP8, *B*. *megaterium* MPP7, and *P*. *putida* MPP18) led to a higher expression of *SOS1* and *SOS4* genes, both of which were associated with an increase in RWC and photosynthetic pigmentation [270]. A PGPB can regulate the exchange of macro- and micronutrients, in addition to reducing the accumulation of Na^+^ and Cl^−^ ions. First, a number of microbial processes have been shown to improve plant access to these nutrients, including Pi (inorganic phosphate) solubilization and siderophore production [160,283]. Secondly, PGPB inoculation can increase protein phosphatases (associated with Pi solubilization). Researchers have reported that *P. simiae* AU treatment increased the presence of VSP (vegetative storage protein) in soybean plants under salinity stress, the enzymatic pathway involved in acid phosphatase activity [278]. This affected the plant’s ability to combat oxidative stress by influencing acid phosphatase activity in the lettuce plants colonized by the *P. mendocina, Palleroni* strain [285]. In addition, microbes can also reduce the uptake of toxic ions by plants by producing ESP, as these compounds act as a physical barrier around the root system, thereby reducing the impact of ion toxicity [159,283,286]. As ESP bind cations, including Na^+^, they decrease the ability of toxic ions to be absorbed by plants, thereby reducing salinity stress (Figure 3) [60,159,160,286]. ESP-producing bacteria improved the wheat growth parameters and altered the nutrient uptake by improving the Na^+^ concentration and boosting K^+^ and Ca^2+^ absorption in plants affected by salinity [246,287]. Similarly, ESP-producing rhizobacteria also revealed their importance in alleviating the salt stress conditions in several crops, such as wheat, soybean, pistachio, or alfalfa [264,279,288,289].

## 8. Drought and Salt-Induced Stress-Responsive Gene Regulation

Plants that exhibit induced systemic tolerance may be influenced by PGPR-mediated stress-responsive genes. When inoculated with *P. polymyxa*, *Arabidopsis* expresses certain genes, including *BAB18* (encoding *LEA* proteins) and *ERD15* (encoding the early response to dehydration) [290]. The inoculation of cucumber plants with a consortium of bacteria, including *B. subtilis, B. cereus*, and *Serratia* sp., has been shown to increase drought resistance by preserving photosynthetic activity through inhibition of the downregulation of *APX* genes and the RuBisCO large and small subunit genes, *rbcL* and *rbcS*. In bean plants treated with *R. etli,* a macroarray analysis of sequence tags revealed an increase in expression of the trehalose-6-phosphate synthase gene, which regulates nitrogen and carbon metabolism [214]. Under water-scarce conditions, *B. licheniformis* can cause the overexpression of several stress-responsive proteins in pepper plants such as early nodulin, adenosine kinase, dehydrin-like protein, *S*-adenosylmethionine synthetase, and vacuolar H^+^-ATPase. Similar effects have been shown by *A. brasilense* and *B. amyloliquefaciens* on wheat leaves, which cause the upregulation of *APX1*, S-adenosylmethionine synthase, and a heat shock protein gene [291]. Sugarcane has been shown to be responsive to ABA-dependent signaling genes activated by *G. diazotrophicus* [202], and mungbean plants can be stimulated by *P. aeruginosa* to enhance the expression of *DHN*, *DREB2A*, and *CAT1* [213]. In *Arabidopsis*, under salinity stress conditions, *Enterobacter* sp. induces the expression of salinity stress-responsive genes (*RD29 A*, *RD29 B*, and *RAB18*); regulons of ABA-responsive elements; *DREB2B*; and dehydration-responsive elements, thereby causing ABA-independent activation (Figure 2) [292]. In *A. thaliana, P. chlororaphis* induces the transcription and upregulation of jasmonic acid (JA) marker genes (*PDF-1.2* and *VSP1*), the ethylene-responsive gene (*HEL*), and the SA-regulated gene (*PR-1*) while downregulating the drought signaling response genes [209,293].

## 9. Conclusions and Future Prospects

In plants, abiotic stress, including drought and salinity, causes not only environmental problems but also social and economic ones. The changing environmental conditions are negatively affecting plants, causing lower growth and yields. As abiotic stresses intensify, plant cells produce ROS, which impairs the ecological fitness. The crisis will be exacerbated in the coming decades, threatening plant survival. Antioxidants are recruited by plants to maintain equilibrium between ROS quenching and its generation. The plant’s response to these events is so rapid that it cannot withstand adverse conditions. PGPB promote the plant’s growth by various mechanisms such as phosphate solubilization, nutrient mobilization, production of phytohormones, VOCs, vitamins and modulating the antioxidant machinery, osmotic adjustment, maintaining ion homeostasis, metabolic reprogramming, and modulating biochemical and molecular pathways, regulating drought/salt responsive genes and making the crop more tolerant to drought and salinity stress conditions. Thus, PGPB are a good alternative to conventional fertilizers due to cost-effectiveness, eco-friendliness, and sustainability to increase plant tolerance to multiple stresses, including drought and salinity.

PGPBs that may induce plant resistance to a variety of abiotic stressors, particularly those that resemble field environments, should be investigated for future research projects aimed at promoting sustainable agriculture. For the widespread and efficient use of beneficial microbes, scientists need to embark on field studies and make farmers familiar with the benefits that microbes can have on plant health and soil quality. Furthermore, nanoencapsulation technology has just been invented and can be used in field testing. The approach may be used to protect PGPR against abrupt environmental shocks, increase their distribution, and help regulate microbial release in the rhizosphere/field. Additional research is needed to discover if tripartite “plant–fungal–bacterial” symbioses can generate synergistic effects on plants. These approaches appear promising for drought and saline agriculture in the future. With the above information, it is evident that we are on the right path towards achieving our goal of sustainable food production in a climate that keeps changing. Governments and federal agencies need to promote the utilization of PGPB-formulated biofertilizers as eco-friendly alternatives for crop improvement. More investments should be done by entrepreneurs in biofertilizer companies and allocate financial assistance for start-ups. Additionally, public awareness is needed to show farmers and consumers the benefits of PGPB-based biofertilizers for sustainable agriculture.

## Data Availability

The data presented in this study are available in the article.

## Abbreviations

ABA	Abscisic acid
ACCD	1-aminocyclopropane-1-carboxylate deaminase
APX	Ascorbate peroxidase
CAT	Catalase
EPS	Extracellular polymeric substances
ESP	Exopolysaccharide
GPX	Glutathione peroxidase
GR	Glutathione reductase
H_2_O_2_	Hydrogen peroxidase
HEL	Ethylene responsive gene
HKT	Sodium transporter
IAA	Indole-3-acetic acid
K^+^	Potassium ion
LPX	Lipid peroxidase
MDA	Malondialdehyde
NHX	Vacuolar Na^+^/H^+^ antiporter
POX	Peroxidases
PRP	Proline-rich protein
ROS	Reactive oxygen species
RWC	Relative water content
SOD	Superoxide dismutase
TSS	Total soluble sugar
VOCs	Volatile organic compounds
VSP1	Vegetative storage protein
